# Multi-Site Clinical Evaluation of DW-MRI as a Treatment Response Metric for Breast Cancer Patients Undergoing Neoadjuvant Chemotherapy

**DOI:** 10.1371/journal.pone.0122151

**Published:** 2015-03-27

**Authors:** Craig J. Galbán, Bing Ma, Dariya Malyarenko, Martin D. Pickles, Kevin Heist, Norah L. Henry, Anne F. Schott, Colleen H. Neal, Nola M. Hylton, Alnawaz Rehemtulla, Timothy D. Johnson, Charles R. Meyer, Thomas L. Chenevert, Lindsay W. Turnbull, Brian D. Ross

**Affiliations:** 1 Departments of Radiology, University of Michigan, Ann Arbor, Michigan, United States of America; 2 Departments of Internal Medicine, University of Michigan, Ann Arbor, Michigan, United States of America; 3 Departments of Radiation Oncology, University of Michigan, Ann Arbor, Michigan, United States of America; 4 Departments of Biostatistics, University of Michigan, Ann Arbor, Michigan, United States of America; 5 Centre for MR Investigations, Hull York Medical School, University of Hull, Hull, United Kingdom; 6 Department of Radiology, University of California San Francisco, San Francisco, California, United States of America; Indiana University, UNITED STATES

## Abstract

**Purpose:**

To evaluate diffusion weighted MRI (DW-MR) as a response metric for assessment of neoadjuvant chemotherapy (NAC) in patients with primary breast cancer using prospective multi-center trials which provided MR scans along with clinical outcome information.

**Materials and Methods:**

A total of 39 patients with locally advanced breast cancer accrued from three different prospective clinical trials underwent DW-MR examination prior to and at 3–7 days (Hull University), 8–11 days (University of Michigan) and 35 days (NeoCOMICE) post-treatment initiation. Thirteen patients, 12 of which participated in treatment response study, from UM underwent short interval (<1hr) MRI examinations, referred to as “test-retest” for examination of repeatability. To further evaluate stability in ADC measurements, a thermally controlled diffusion phantom was used to assess repeatability of diffusion measurements. MRI sequences included contrast-enhanced T1-weighted, when appropriate, and DW images acquired at b-values of 0 and 800 s/mm^2^. Histogram analysis and a voxel-based analytical technique, the Parametric Response Map (PRM), were used to derive diffusion response metrics for assessment of treatment response prediction.

**Results:**

Mean tumor apparent diffusion coefficient (ADC) values generated from patient test-retest examinations were found to be very reproducible (|ΔADC|<0.1x10^-3^mm^2^/s). This data was used to calculate the 95% CI from the linear fit of tumor voxel ADC pairs of co-registered examinations (±0.45x10^-3^mm^2^/s) for PRM analysis of treatment response. Receiver operating characteristic analysis identified the PRM metric to be predictive of outcome at the 8–11 (AUC = 0.964, p = 0.01) and 35 day (AUC = 0.770, p = 0.05) time points (p<.05) while whole-tumor ADC changes where significant at the later 35 day time interval (AUC = 0.825, p = 0.02).

**Conclusion:**

This study demonstrates the feasibility of performing a prospective analysis of DW-MRI as a predictive biomarker of NAC in breast cancer patients. In addition, we provide experimental evidence supporting the use of sensitive analytical tools, such as PRM, for evaluating ADC measurements.

## Introduction

An important component in the treatment of primary breast cancer is the use of adjuvant systemic therapy. This allows for the opportunity to provide for a reduction in the risk of recurrence and death [1–5]. In breast cancer patients, randomized studies have found that pre-operative chemotherapy provides a similar survival benefit from a particular treatment regimen which is similar to post-operative therapy [5]. Preoperative therapy is an important approach as it allows for the possibility of down-staging the primary tumor in the majority of women thus improving rates of breast preservation [6,7]. Moreover, preoperative therapy also has an additional benefit of assessing the *in vivo* tumor response to a particular drug regimen. Current evaluation of systemic pre-operative therapies relies on post-surgical assessment of removed tissue [8,9], and pathologic complete response (pCR) has been found to be a powerful surrogate of long-term disease-free survival [6–9]. Thus, it is postulated that a therapeutic regimen that produces higher rates of CR in the neoadjuvant chemotherapy (NAC) treatment setting will also provide for higher rates of long-term cure. Ideally, a patient’s response to NAC should be detected early and noninvasively using imaging to provide quantitative assessment of treatment responsiveness. As more varied, targeted, and effective systemic therapies are developed, this capability could facilitate the individualization of patient care by providing the opportunity to tailor subsequent treatments for a particular patient based on response to the initial treatment.

DW-MR provides the ability to quantify changes in the Brownian motion of water [10] which is capable of detecting subtle changes in the microenvironment of living tissue. The structure within the microenvironment that affects water diffusivity includes tissue cellularity and extracellular volume, especially when changes are monitored early following treatment initiation. Initial application of diffusion characterization of CNS tumors revealed high apparent diffusion coefficient (ADC) values within necrotic regions of tumors [11–13]. These observations were confirmed in subsequent diffusion studies on both human and animal tumors [14–16]; and recently a correlation between tumor cellularity and ADC was demonstrated in a study of glioma patients [17]. These works suggest diffusion has the potential to aid distinction of necrotic from viable tumor. Given that diffusion MRI is sensitive to structure at the cellular level, it has the potential to detect and quantify cellular changes that occur in response to successful therapeutic intervention. Moreover, it is reasonable to expect such changes would be measurable prior to macroscopic changes in mass, size, or morphology since removal of debris occurs relatively slowly. The consistent observation of high diffusion in necrotic tissue relative to solid tumor suggests a positive therapeutic effect should register as an increase in diffusion values relative to untreated tumor. Indeed, this has been the pattern observed by several groups using a variety of tumor models and anti-cancer treatments [18–20]. In our experiments with an intracranial rodent glioma model, we observed a 50–100% increase in solid tumor diffusion values following treatment with a chemotherapeutic agent [18–21]. Changes were measurable within two days, peaked within 6–8 days following treatment, and persisted until tumor re-growth shifted ADC back to pretreatment levels. Qualitatively similar findings have been reported using multiple murine tumor models (including breast tumors [22]) and different therapies [18–25]. Results of these studies suggest that quantitative water diffusion measurement/imaging offers potential for early assessment of anti-neoplastic treatment response. Through our own research efforts and those of other laboratories DW-MR is proving to be capable of evaluating treatment response in both preclinical and clinical settings as an early biomarker of subsequent tumor response [26–31]. In preliminary pilot studies, DW-MR was recently reported to show promising results for early response assessment in breast cancer patients [32–35] thus DW-MR appears to offer substantial potential for making substantial inroads into the goal of predicting clinical treatment response early using imaging metrics.

**Fig 1:**
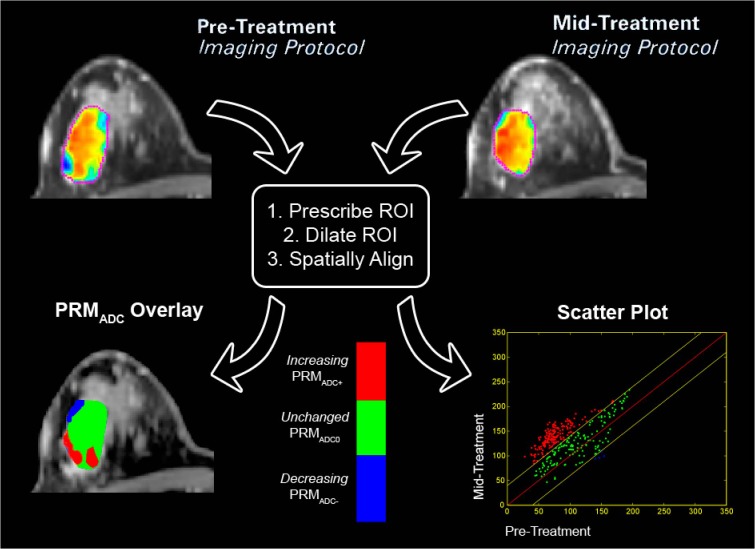
Pictorial representation of the PRM analytical process on ADC maps. Regions of interest (ROI) are prescribed on the pretreatment anatomical images. ROI are then dilated to encompass neighboring tissue around tumor. Control points are automatically distributed throughout the new ROI, where three-five control points must be user defined. Diffusion–weighted MRI data undergoes co-registration to pretreatment anatomical image. Registered pre and mid-treatment ADC maps are used to generate a three-color overlay representing regions in which tumor ADC values significantly increased (red voxels), significantly decreased (blue voxels) or remain unchanged (green voxels). This data can also be presented in a scatter plot and percentages assigned to the three defined ADC regions, allowing quantitative assessment of overall changes in tumor ADC values.

Much of the work to date has involved the use of summary statistics for evaluating therapeutic response using ADC measurements in tumor. Although an increase in water diffusivity as measured by DW-MR has been shown to reflect improved killing of tumor cells [36], spatial heterogeneity in the tumor response to treatment has been shown to attenuate the sensitivity of ADC as determined by whole-tumor statistic (e.g. mean) [37,38]. In a recent study of a population of patients with primary CNS tumors, we prospectively compared tumor ADC at 3 weeks after initiation of therapy with pretreatment images to quantify therapy-induced changes in ADC [37,38]. To account for regional variations in response, two image datasets from before and after treatment were co-registered [39] and analyzed to yield parametric response maps (PRM) as illustrated in Fig 1 [40–42]. These maps present a color overlay of therapeutic induced ADC changes within the tumor, where different regions within the tumor are stratified based on increasing (red), decreasing (blue), or stable (green) ADC values. PRM response metrics were found to correlate better with radiographic response at 10 weeks [36–38,43,44] and overall survival [43] than either histogram measured mean ADC changes or early volumetric changes.

In this study, we obtained DW-MR data from both a single site and from a multi-center prospective clinical trial in which individuals with primary breast cancer treated with NAC were enrolled. To monitor for treatment-induced alterations in tumor cellularity, serial DW-MR scans were acquired at baseline (prior to treatment) and at again at 1–2 time intervals following the start of therapy but before treatment conclusion. Clinical outcomes data was used to assess the utility of PRM, a voxel-based analytical technique and percent change in mean histogram values, as applied to serial ADC maps for their predictive capability as imaging biomarkers for early assessment of clinical treatment response. In addition, a subset of patients from a single-center prospective trial was used to determine the repeatability threshold of ADC measurements by acquiring short interval (<1hr) serial DW-MR examinations to delineate instrumentation noise and ADC measurement variability using a thermally-controlled diffusion phantom [45–47]. We report that ADC measurements can be reliably obtained serially in breast cancer patients undergoing NAC and that PRM analysis appears to provide for improved sensitivity over histogram-based mean diffusion changes for early response assessment.

## Materials and Methods

### University of Michigan (UM)

#### Ethics Statement

This study was approved by the University of Michigan Institutional Review Board. Subjects with newly diagnosed breast cancer were enrolled on a protocol of intra-treatment MRI (IRB: HUM00003392, Patient Recruitment: 2006–2013). Written informed consent was given by the patients for their information to be used for our research. The University of Michigan clinical trial (UM) consisted of short interval MRI examination, approximately within a 30 minute interval, which were acquired on 13 subjects within 7 days prior to therapy. Eleven of these subjects were used for subsequent analysis as one patient opted out of the study and another was not utilized due to an indeterminate clinical outcome, had an additional MRI examination performed at 8–11 days post-treatment initiation. Treatment consisted of NAC consisting of doxorubicin (60 mg/m^2^) plus cyclophosphamide (600 mg/m^2^) administered every 2 weeks for a total of four cycles, followed by paclitaxel administered every 2 weeks for an additional four cycles of NAC. Clinical response was assessed by palpation following the conclusion of the first treatment cycle.

MRI scans were performed on a 3T Philips Achieva MRI system (Best, the Netherlands) using a seven-channel phased-array breast coil. Each interval exam included a gadolinium–diethylenetriamine pentaacetic acid (Gd-DTPA; Bayer HealthCare Pharmaceuticals, Wayne, NJ)–enhanced three-dimensional fast field echo T_1_-weighted sequence (FOV, 240×240 mm; matrix, 240×240; slice thickness, 1 mm; slices,160; TR/TE/inversion time [TI], 9.9:4.6:1040 milliseconds; turbofactor 200), and a diffusion-weighted (DW), single-shot, spin-echo, echo-planar imaging (EPI) series (FOV, 270×270 mm; matrix, 205×205; slice thickness, 4 mm; slices, 24; TR/TE, 2789/59 milliseconds; b factor 0 and 800 sec/mm^2^) with diffusion sensitization along three orthogonal directions. DWI was acquired prior to Gd-DTPA administration. All images were acquired with a SENSitivity Encoding (SENSE) acquisition scheme [22]. The SENSE factor for all images was 2, except diffusion scans which had a SENSE factor of 3.9. The total duration of this acquisition protocol (including patient setup) was approximately 35 to 60 minutes, with DW-MR scans requiring approximately 2 minutes of scan time.

### University of Hull, England and NeoCOMICE

Courtesy of the University of Hull, UK was a single-site study (Hull) consisting of 27 de-identified image data sets with clinical outcomes provided, of which 13 were used in this study. An additional 26, of which 14 were used, were provided from the Cancer Research UK funded multisite UK clinical trial NeoCOMICE (UKCRN ID 5828) ISRCTN42613663. Both the University of Hull and NeoCOMICE had IRB approvals from their respective institutions for these trials. Written informed consent was given by the patients for their information to be used for our research. All data received by UM from Hull and NeoCOMICE was anonymized and de-identified prior to arrival. The remaining datasets were excluded from the study due to incomplete scans, missing first or second post-treatment data, no outcome results or extensive image artifacts. For both datasets subjects were enrolled who were newly diagnosed with breast cancer. Treatment primarily consisted of epirubicin (90 mg/m^2^) and cyclophosphamide (600 mg/m^2^) administered at 3 week intervals. MR scans were performed approximately 8 days prior to treatment and 7 days and again 35 days post-treatment initiation. Therapeutic outcomes were determined using RECIST 1.1 response criteria from assessment of tumor volumetric changes in MR images from pretreatment to the time of therapy completion [48].

MRI scans were performed on both 1.5T and 3.0T MRI systems utilizing dedicated bilateral breast coils [49]. Each interval exam included Gd-DTPA-enhanced T_1_-weighted sequence (parameters) and a DW, single-shot, dual spin-echo, EPI sequence acquired axially with a water-only excitation (TR/TE, 4000/74 ms (3.0 T) or 4000/98 ms (1.5 T); FOV, 340×340 mm; matrix, 128×128; slice, 5 mm; gap, 1 mm; 10 averages; one b_0_ acquisition, and three b = 700 s/mm^2^ acquisitions applied in orthogonal directions. DW-MR scans were acquired in 2 min 40 sec.

### Thermally-Controlled Diffusion Phantom

We employed a thermally-controlled diffusion phantom, developed by our group, to assess the repeatability of ADC measurements and the impact of instrumentation noise to these measurements. The phantom used in this study has been previously described [45,50]. Briefly, the phantom consisted of 50 mL polypropylene conical tube inserted in a 1000 mL polypropylene wide-mouth jar. The 50 mL tube was filled with distilled water and placed in the 1000 mL jar which was filled with crushed ice and water. The water within the tube equilibrated to 0°C within 30 minutes of insertion into the jar. DW images were acquired on a 3T Philips using a 8-channel head coil using a single-shot, spin-echo, echo-planar imaging (EPI) series (FOV, 270×270 mm; matrix, 205×205; slice thickness, 4 mm; slices, 24; TR/TE, 2789/59 milliseconds; b factor 0 and 800 sec/mm^2^) with diffusion sensitization along three orthogonal directions.

### Image Analysis

All MRI data were transferred to a PC, interpolated to a matrix of 256x256, and analyzed using in-house software developed in MATLAB (The MathWorks, Inc., Natick, MA). The product of the three orthogonal DW-MR images exhibits strong sensitivity to diffusion with no dependence on structural directionality in the tissues. This isotropic feature was crucial for following serial changes in water diffusivity, quantified as the apparent diffusion coefficient (ADC), without confounding effects due to tissue orientation. ADC maps were calculated using the following equation:

$$\text{ADC= ln}\frac{{\text{S}}_{{\text{b}}_{\text{0}}}}{{\text{S}}_{{\text{b}}_{\text{800}}}}/\left({\text{b}}_{\text{800}}{\text{-b}}_{\text{0}}\right)$$ 1

where $S_{b_{0}}$ and $S_{b_{800}}$ are the signal intensities acquired at low and high diffusion sensitivity, respectively, b_0_ and b_800_ are the low and high b-values in units of s/mm^2^, respectively, and ADC is the apparent diffusion coefficient obtained using b_0_ and b_800_. Subsequent to image registration, contours were manually drawn by a MRI breast radiologist over tumors as delineated on contrast-enhanced T_1_-weighted images. From the region-of-interest (ROI) tumor volume and mean ADC were assessed at each interval exam.

### Parametric Response Map (PRM)

Presented in Fig 1 is a schematic representation of PRM work flow. Image registration of the mid-treatment ADC map (homologous image) to the pre-treatment ADC map (reference image) was performed first using a rigid body registration to account for spatial repositioning. As a consequence of the soft breast tissue large deformation may occur during serial examinations. A deformable algorithm that employed thin plate splines was used to account for deformation of the breast tumor [51]. To minimize processing time while increasing accuracy of the deformable registration process, image alignment was performed only on the prescribed ROI. For both rigid and deformable registration mutual information was used as an objective function and simplex as an optimizer [39,52]. Approximately 30 equidistant control points were automatically positioned within the reference image, where 5 of these points were manually selected. The remaining control points were automatically aligned, resulting in an approximate 10 minutes of computational time. Subsequent to image registration, individual voxels, the smallest unit of volume, were classified based on the extent of change in the ADC value. This was performed by calculating the difference between ADC values (ΔADC = mid-treatment ADC–pre-treatment ADC) for each voxel within the tumor ROI. Voxels yielding ΔADC greater than a predetermined threshold of 0.45x10^-3^mm^2^/s (described below) were coded red (i.e. Red: ΔADC > 0.45x10^-3^mm^2^/s), voxels with values less than -0.45x10^-3^mm^2^/s were coded blue (i.e. Blue: ΔADC < 0.45x10^-3^mm^2^/s), and all other voxels were coded green (i.e. Green: -0.45x10^-3^mm^2^/s **≤** ΔADC ≤ 0.45x10^-3^mm^2^/s). Global PRM measures were calculated by normalizing the sum of all voxels within a classification by the total tumor volume. The nomenclature of these measures are PRM_ADC+_ for the relative tumor volume with increasing ADC, PRM_ADC-_ for the relative tumor volume with decreasing ADC, and PRM_ADC0_ for the relative tumor volume with unchanged ADC.

We empirically calculated the thresholds that designate a significant change in ADC within a voxel from the 13 subjects who underwent pre-treatment serial MRI examination within a short time interval (~30 minutes between examinations). For each subject, tumors were manually contoured and spatially aligned as described above such that each tumor voxel consisted of an ADC pair. We then determined the 95% confidence intervals (CI) from the resulting linear least-squares fit of the joint density histogram (illustrated in Fig 1). The mean of all 95% CIs was used as the PRM threshold for therapeutic response assessment of the multisite data.

### Data and Statistical Analysis

To illustrate the importance of image registration to account for spatial heterogeneity in a tumor volume we determined the 95% confidence interval of tumor ADC differences for each subject. Short interval ADC measurements, “test-retest”, were evaluated by calculating the mean and the standard deviation of ADC values over the entire tumor volume for each subject. As an approximation, the error associated with the difference in the serial mean ADC values was determined by propagation of error, σ_Δ_^2^ = σ_A_^2^+ σ_B_^2^ where σ is the standard deviation and A, B and Δ indicate the first examination, second examination and difference between examinations, respectively. The 0.975 quantile of the difference in mean ADC values within a subject tumor was then determined by the following expression, 1.96xstandard deviation. Differences in age and initial tumor volume between accrual sites were determined using an unpaired 2-tailed Student’s t-test. Differences in tumor grade between sites were determined using a Likelihood Ratio test. Due to the relatively small number of subjects in each of the studies, patient’s designated complete response (CR) and partial response (PR) were pooled into a single classifier called responders and stable disease (SD) and progressive disease (PD) patients were classified as non-responders. The percent change in mean ADC and PRM measures were assessed between responders and non-responders by an unpaired 2-tailed Student’s t-test at time points 3–5 days, 8–11 days and 35 days. Finally, receiver operating characteristic (ROC) curve analysis was performed to determine the predictive potential of the percentage change in ADC and PRM parameters with subject clinical response. Data are presented as mean±SEM, unless stated otherwise. All statistical computations were performed using a statistical software package (IBM SPSS Statistics, Armonk, NY), and declared statistically significant at the two-sided 5% comparison-wise significance level (p < 0.05).

The raw data used in the final analyses has been provided as a table (S1 Table). This table contains anonymized numerical summary statistics, at the individual voxel and subject level of ADC and delta-ADC values from within the tumor ROIs for each subject/time point. Furthermore, while informed consent and HIPA regulations apply to this clinical trial data set which places limits on providing the original image data, interested parties seeking to access the original image data should contact the communicating author to discuss and complete the required institutional Material Transfer Agreement documentation.

## Results and Discussion

### Test-Retest

**Fig 2:**
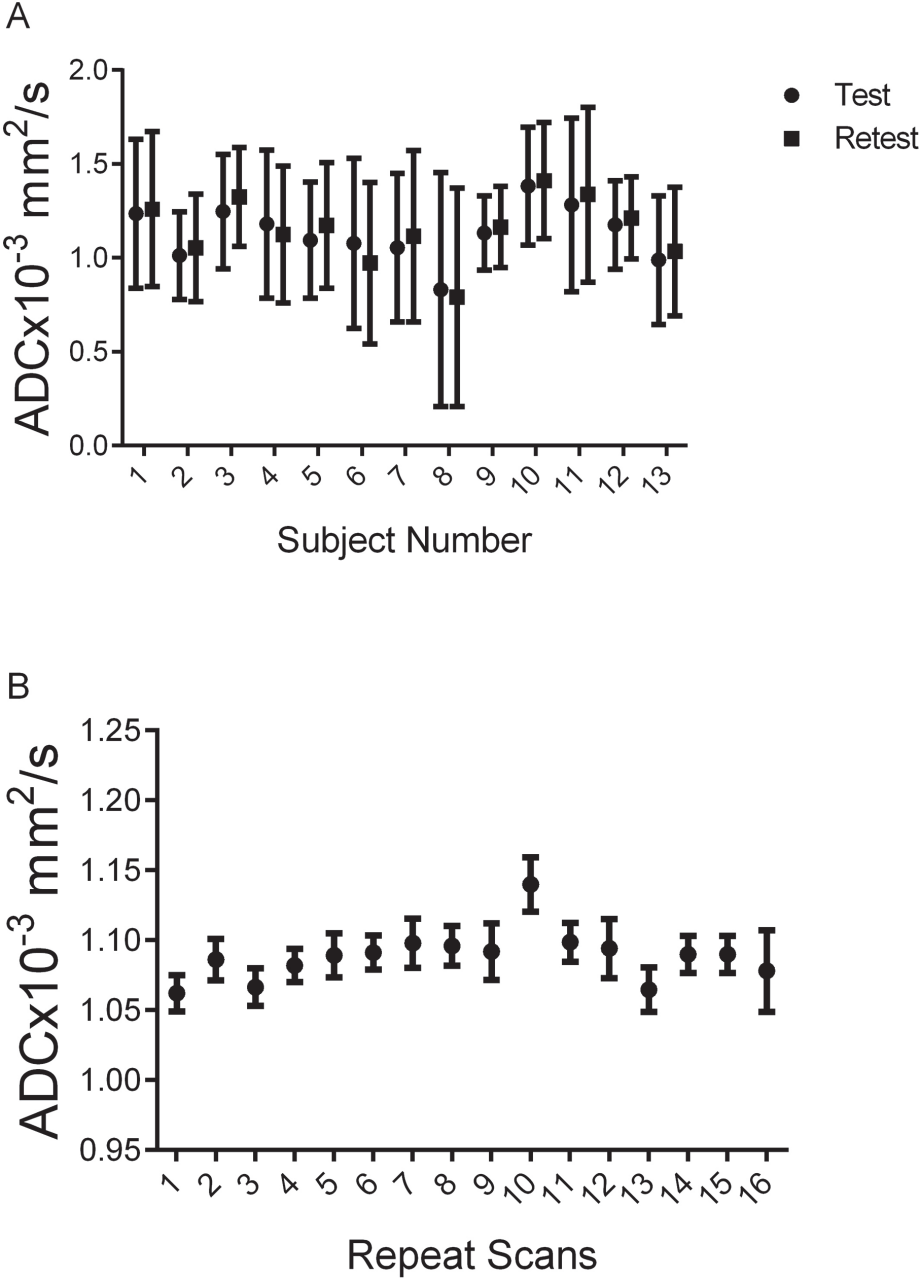
ADC Results from test-retest human breast and phantom studies. Presented are summary plots of mean ADC values from (A) the test-retest of breast tumors from individual subjects accrued at the UM and (B) the repeatability analysis using a thermal-controlled diffusion phantom (i.e. ice water phantom [45,50]). Data is presented as the mean±standard deviation.

To determine variations in ADC measurements associated with instrumentation noise as well as provide a means of establishing thresholds for the PRM approach, MRI examinations were obtained in short intervals on 13 breast cancer patients accrued at the UM. Fig 2A demonstrates the test-retest results for these 13 subjects. Mean values in tumor ADC were bounded between 0.5 and 1.5x10^-3^mm^2^/s with standard deviations observed as high as 0.6x10^-3^mm^2^/s. In a comparative study, we performed a repeatability experiment where on 16 separate occasions ADC measurements were acquired from a thermal-controlled diffusion phantom that consisted of liquid water at near freezing temperature [45,50]. Here, ADC values were consistently measured around the literature value of 1.1x10^-3^mm^2^/s [53] with the mean in the standard deviations at 0.02x10^-3^mm^2^/s (Fig 2B). As this was a homogeneous water phantom, the observed standard deviations in the ADC measurements are directly attributed to instrumentation noise. We postulate that the large discrepancy in the standard deviations of the ADC values between the breast tumors and phantom was attributed almost entirely by tumor heterogeneity.

**Fig 3:**
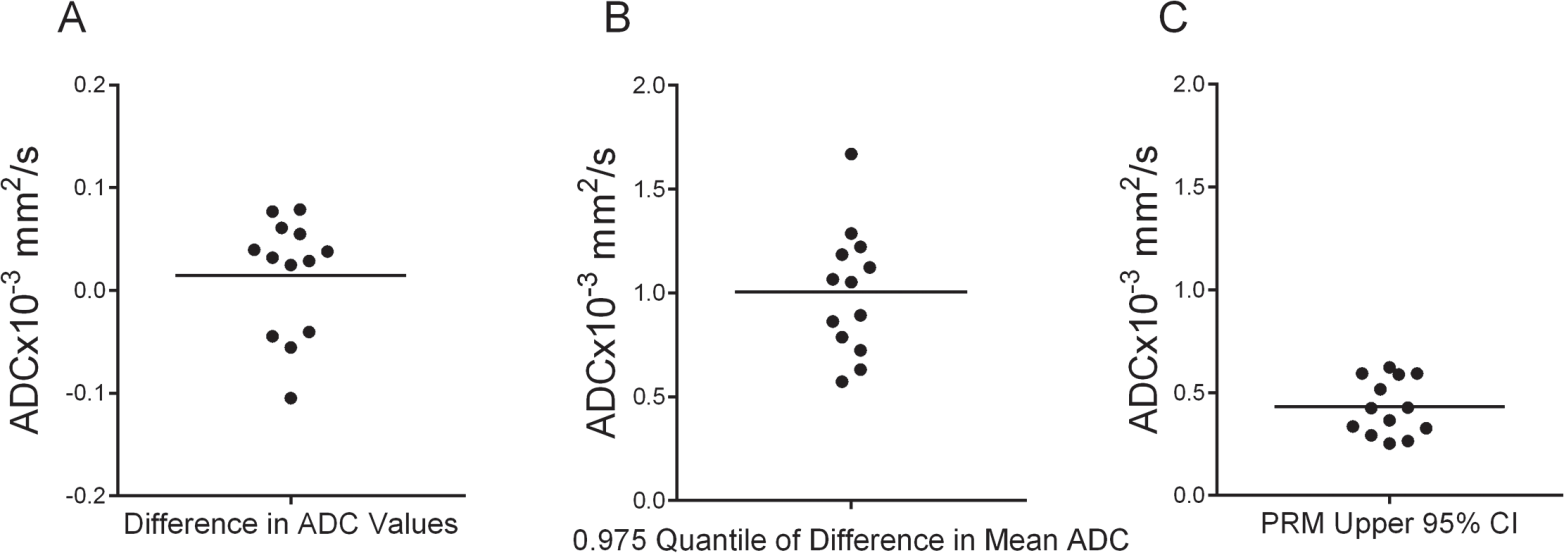
Evaluation of ADC variability in test-retest data. Presented are scatter plots of (A) the difference in mean ADC tumor values, (B) the 0.975 quantile of the difference in mean ADC tumor values, and (C) the PRM 95% CI generated from the fit of the joint density histogram of voxels from spatially aligned serial ADC tumor maps. The 0.975 quantile of the difference in mean ADC values was determined by propagating the error (standard deviation) and calculating the 0.975 quantile as 1.96xstandard deviation. Each dot represents a single patient and the large line the mean of the entire group.

As seen in Fig 3A, the difference in the mean ADC tumor values between serial examinations varied by less than ±0.1x10^-3^mm^2^/s. By propagating the error, we acquired an estimate of the 0.975 quantile of the difference in the mean ADC tumor values for each subject. Here we found the mean 0.975 quantile was about 1x10^-3^mm^2^/s for all subjects, again attributed mostly to large variations in tumor tissue rather than instrumentation noise (Fig 3B). The 95% confidence interval obtained from the linear least-squares fit of the joint density histogram of spatially aligned serial ADC maps (illustrated in Fig 1) was found to be approximately half (mean value of 0.45x10^-3^mm^2^/s with a range of 0.25 to 0.62x10^-3^mm^2^/s) of what was observed by simply propagating the error from the whole-tumor estimates. In the absence of any physiological changes in the tumor that may adversely affect tumor ADC, only instrumentation noise would cause variability in the ADC measurement from serial maps perfectly aligned. As this is not the case, slight imperfections in the image registration have occurred. Nevertheless, the value determined from the joint density histogram is consistent with previously published results [38,54,55]. All subsequent PRM analyses were performed using this determined value (i.e. ±0.45x10^-3^mm^2^/s).

### Subject Characteristics

Thirty-nine subjects with newly diagnosed primary breast cancer were included in this analysis, accrued as part of three separate clinical trials: 11 from UM, 13 from Hull and 14 from the multicenter UK trial NeoCOMICE. A summary of patient characteristics is provided in Table 1. All subjects underwent NAC for multiple cycles following their own respect treatment regimen. Controlling for multiple comparisons, Hull subjects were found to be significantly older in age than UM subjects (p = 0.003). Although there was some disparity in patient age, there were no significant differences in tum or grade or initial tumor volume between accrual sites.

**Table 1:** Patient Characteristics.

**ID**	**Site**	**Age**	**Type**	**Initial Tumor Volume (cm3)**	**Grade**	**Outcome**
1	UM	59	ductal	10.9	3	PR
2	UM	48	ductal (apocrine)	3.2	3	PR
3	UM	47	ductal	25.8	2	PR
4	UM	41	ductal (apocrine)	12.2	2	SD
5	UM	24	ductal	11.6	3	CR
6	UM	64	ductal	5	3	PR
7	UM	41	ductal	13.3	2	SD
8	UM	41	ductal	4.2	3	CR
9	UM	43	ductal	12.8	2	SD
10	UM	37	ductal	1.6	1	PR
11	UM	46	ductal	4.4	3	PD
12	Hull	51	NST	15.7	2	SD
13	Hull	66	NST	5.5	3	PR
14	Hull	54	Ductal	16.9	3	PR
15	Hull	62	NST	28	3	PR
16	Hull	69	NST	4.3	3	PR
17	Hull	53	Ductal	20.5	3	PR
18	Hull	55	Ductal	18.3	3	SD
19	Hull	53	Ductal	61	2	PR
20	Hull	46	NST	54.8	3	PR
21	Hull	57	Ductal	36.8	2	PR
22	Hull	58	NST	6.3	3	SD
23	Hull	65	Mucinous	12.1	2	PR
24	Hull	59	Ductal	11.5	2	PR
25	NeoComice	41	Ductal/no special type	166.6	2	SD
26	NeoComice	63	Ductal/no special type	21.5	3	PD
27	NeoComice	62	Ductal/no special type	5.6	3	SD
28	NeoComice	62	Ductal/no special type	9.8	3	PR
29	NeoComice	47	Ductal/no special type	14.6	2	SD
30	NeoComice	48	Ductal/no special type	8	2	SD
31	NeoComice	48	lobular	181	2	SD
32	NeoComice	50	Ductal/no special type	4.6	3	SD
33	NeoComice	34	Ductal/no special type	7.2	3	SD
34	NeoComice	44	Ductal/no special type	27.4	2	SD
35	NeoComice	56	Ductal/no special type	35.7	3	SD
36	NeoComice	53	lobular	11.2	2	PR
37	NeoComice	57	Ductal/no special type	9.9	2	PD
38	NeoComice	39	Ductal/no special type	45.2	3	PR

Note: Outcomes are based on RECIST with the convention: CR for complete response, PR for partial response, SD for stable disease and PD for progressive disease.

### Therapeutic Response by DW-MRI

**Fig 4:**
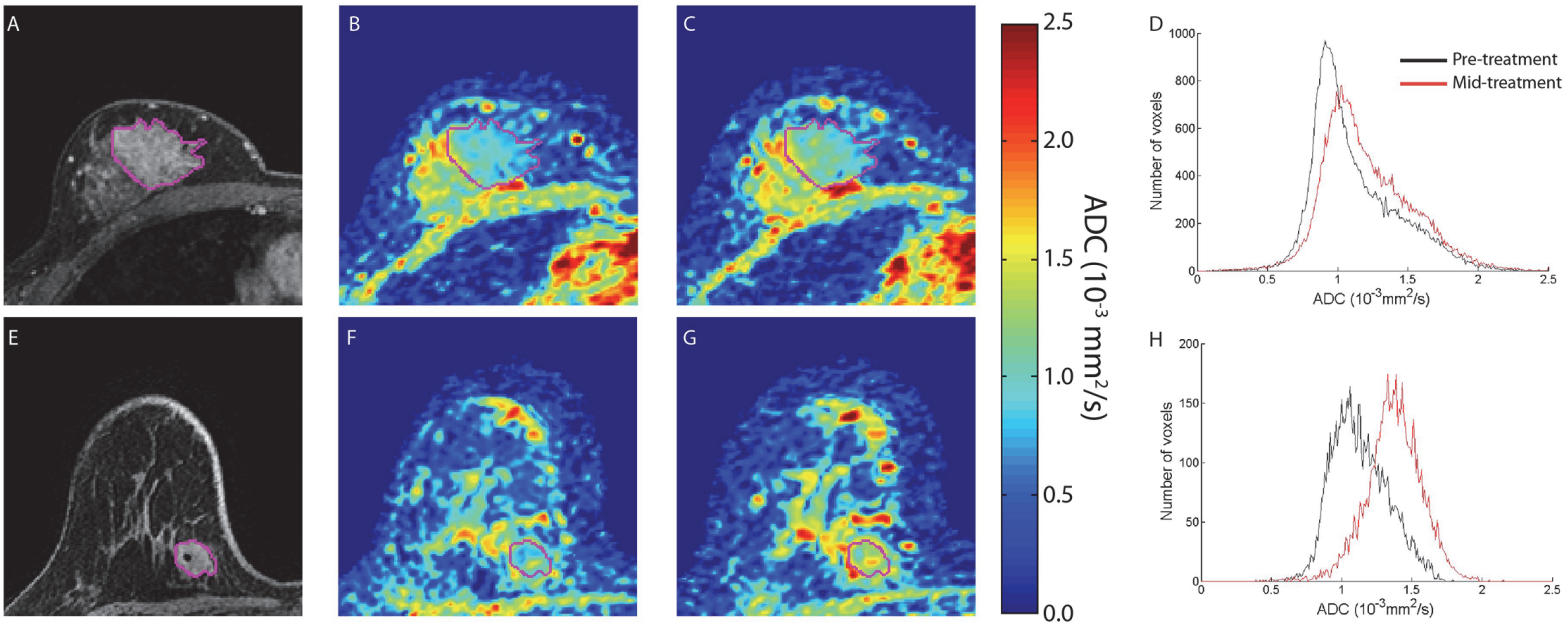
Therapeutic-induced changes in breast tumor ADC values. MRI images are depicted for non-responding (top row) and responding (bottom row) patients treated for breast cancer. (A) and (E) T1-weighted gadolinium enhanced, (B) and (F): pre-treatment ADC maps, (C) and (G): ADC maps at 8–11 days after treatment initiation, D) and (H): Histograms of ADC values in the tumor pre-treatment and post-treatment initiation. Tumor is delineated from surrounding healthy tissue in the individual images by the purple line.

Contrast-enhancing images and ADC maps pre and mid-treatment are presented for UM accrued patients identified by palpation following one cycle of treatment as SD and CR (Fig 4 top and bottom rows, respectively). The representative ADC maps pre-treatment clearly identify the heterogeneous distribution of ADC values throughout the tumor (Fig 4B and 4F). Following a therapeutic intervention, a negligible change in the tumor ADC at serial time points was observed in the patient with the stable disease. Mean ADC values were found to be 1.1x10^-3^ mm^2^/s and 1.2x10^-3^ mm^2^/s pre and mid-treatment, respectively, resulting in a percent change in ADC of 8% (Fig 4D). Only a 6% decrease in tumor volume was observed in the SD patient. For the CR patient, the histogram of the mid-treatment ADC values, as seen in Fig 4, shifted to higher ADC values. In fact, mean ADC increased by 21% from 1.1x10^-3^ mm^2^/s and 1.4x10^-3^ mm^2^/s pre and mid-treatment, respectively (Fig 4H), whereas the tumor volume had only decreased by 2%.

**Fig 5:**
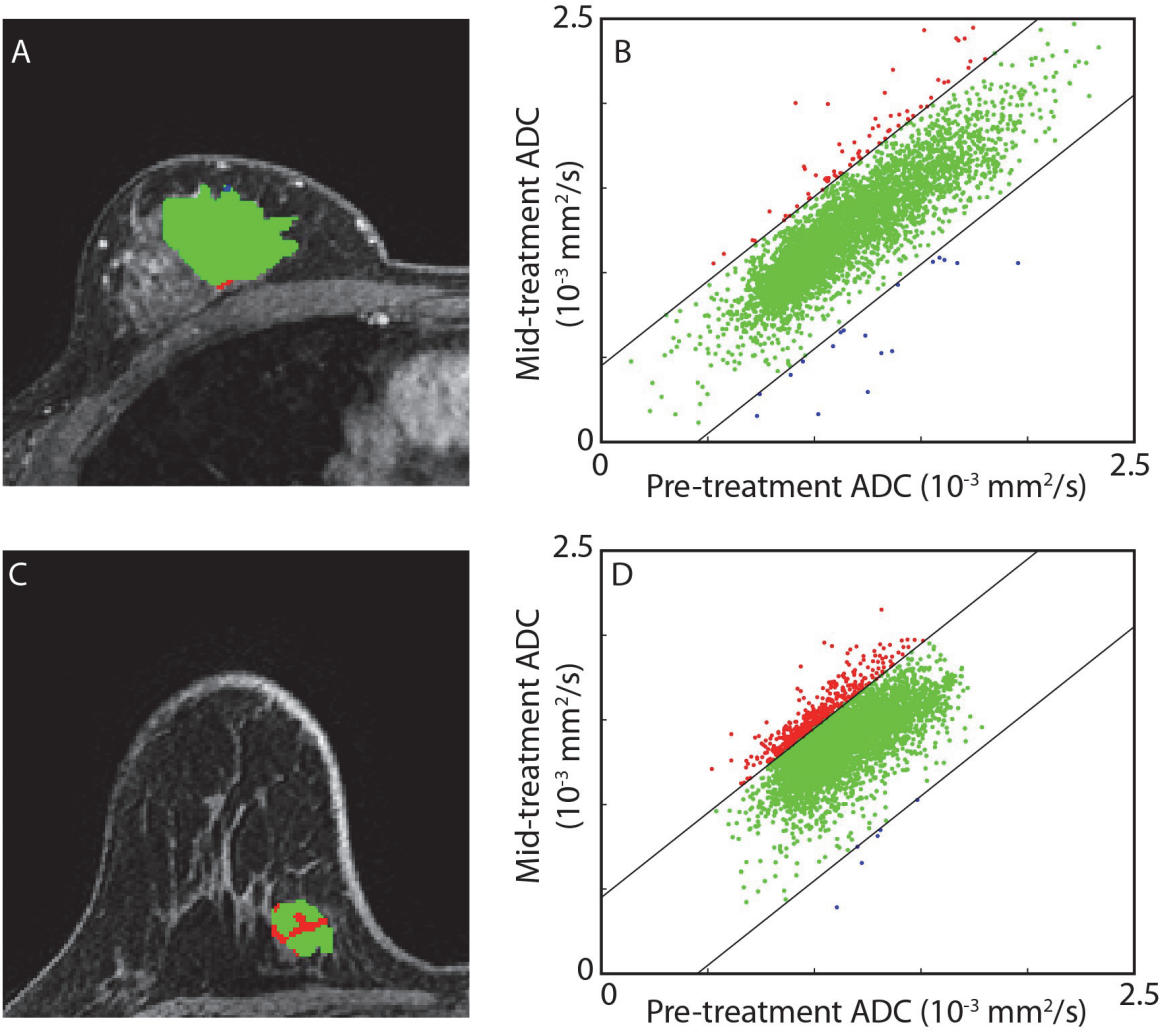
Evaluation of PRM_ADC_ as a response metric. Parametric response maps (A) and (C) and corresponding scatter plots (B) and (D) of post- versus pre-treatment ADC values are presented for a representative non-responder (top row) and responder (bottom row). The joint density histogram from the non-responder demonstrated a negligible shift resulting in a PRM_ADC+_, i.e., the relative tumor volume with significantly increasing ADC values, of 1.8%. In contrast, a substantial shift in the histogram was observed for the responder (PRM_ADC+_ of 12.8%).

Application of the PRM technique on serial ADC maps identified substantially less tumor volume with increasing ADC values mid-treatment in the SD patient as compared to the CR patient (Fig 5). For the SD patient, only 1.8% of the tumor volume was found to generate increasing ADC values that were beyond the 95% confidence interval (±0.45x10^-3^mm^2^/s). This suggests that a large segment of the tumor volume was unresponsive to the therapeutic intervention. For the CR patient, up to 12.8% of the tumor was designated by PRM as demonstrating an increase in ADC beyond the 0.45x10^-3^mm^2^/s threshold. Responsive tissue within the tumor is clearly identified with the PRM_ADC_ map as red voxels, suggestive of a reduction in tumor cellularity in response to an effective therapy. Increasing values in PRM_ADC+_ has been shown to correlate with cell kill in preclinical models of brain tumors and metastatic cancer to the bone [27,36].

**Fig 6:**
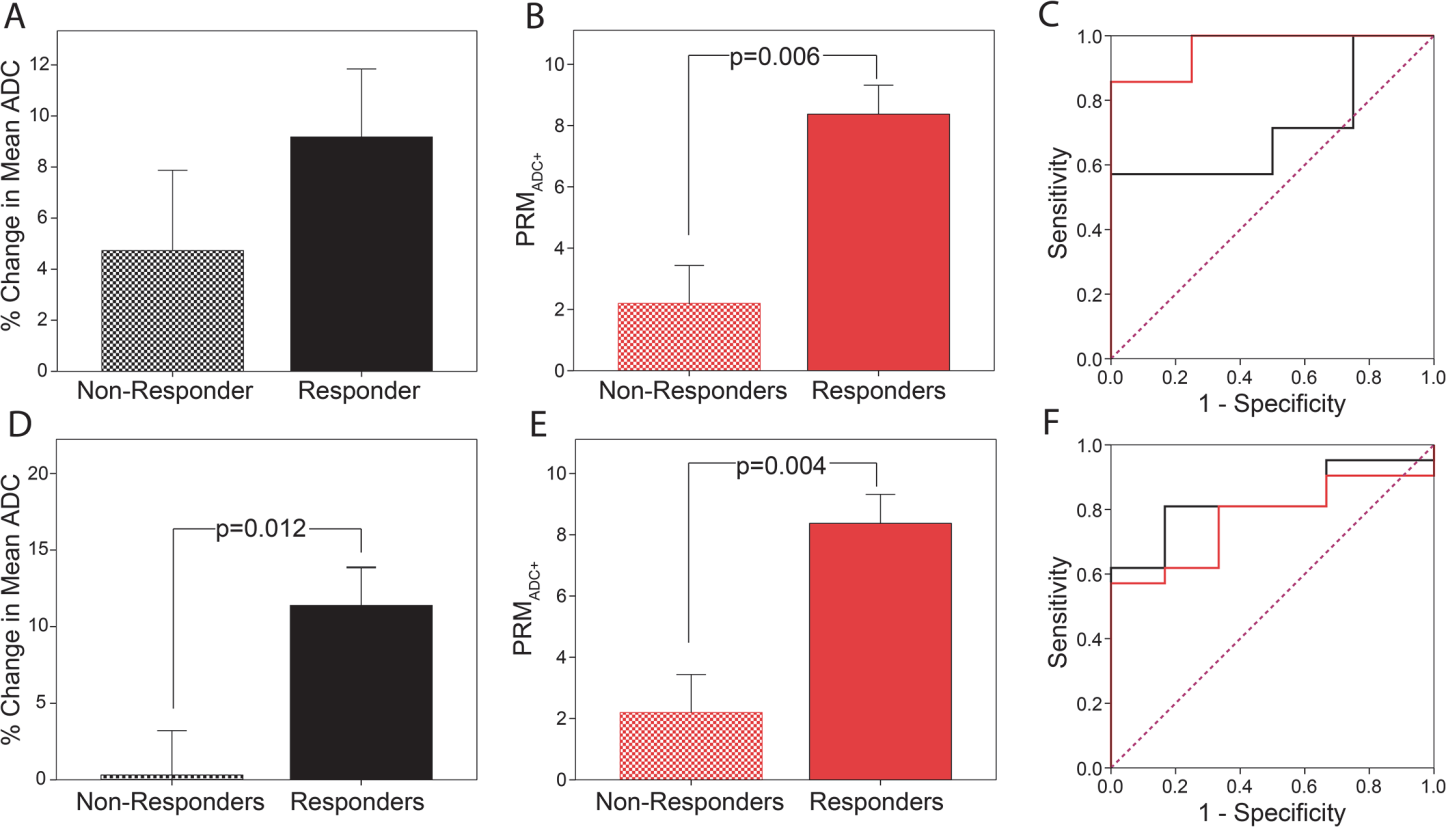
DW-MRI results at interval MRI examinations. Presented are results from MRI data acquired at (A-C) UM (8–11 days) and (D-F) NeoCOMICE (35 days). The analyses include the percent change in mean ADC values and (B, E) PRM values of cancer patients diagnosed as responders and non-responders at three different interval MRI examinations as well as (C,F) ROC analysis for both readouts. Statistical significance was assessed at p<0.05. Data acquired at 8–11 days was obtained at the University of Michigan, where other interval data was acquired as part of a UK clinical trial.

MRI examination time intervals varied between sites. As such, we evaluated PRM and percent change in ADC from histogram analysis at 3–5 days (Hull and NeoCOMICE), 8–11 days (UM) and 35 days (Hull and NeoCOMICE) post-treatment initiation along with ROC analysis of several individual imaging-based biomarker metrics (Fig 6). For the analysis, the response groups were pooled such that CR and PR were classified as responders and SD and PD were classified as non-responders. Percentage change determined from ADC histogram analysis was not significant for patients classified as responders versus non-responders at either the 3–5 day interval (10.6±4.5% and 3.3±2.2%) or the 8–11 day interval (9.1±2.7% and 4.7±3.1%) (Fig 6A). However, the percent change in the mean tumor ADC was found to be significantly higher in responders at 35 days post-treatment initiation versus non-responders (11.3±2.5% for responders and 0.3±2.9% for non-responders, p = 0.012) (Fig 6D). PRM_ADC-_ produced no significant difference between responders and non-responders at 3–5 days (3.2±0.6% and 3.5±1.2%), 8–11 days (3.8±1.4% and 2.1±1.6%) and 35 days (6.6±2.1% and 8.8±3.4%). PRM_ADC+_ at 3–5 days was similar between groups (8.3±1.9% for responders and 8.6±3.2% for non-responders). Differences in the PRM_ADC+_ metric became evident at as early as the 8–11 day examination time point (8.4±0.9% for responders and 2.2±1.2% for non-responders; p = 0.006) with responders (Fig 6B). Furthermore, PRM_ADC+_ was also found to be predictive of response at the 35 day interval with responders having a PRM_ADC+_ of 17.4±2.8% which was significantly higher in value from the PRM_ADC+_ of the non-responder group (7/4±1.5%, p = 0.004) (Fig 6E). For the 8–11 day data, ROC analysis revealed an AUC value of 0.964 (p = 0.01) versus 0.714 (p = 0.26) for PRM_ADC+_ and percent change in mean histogram ADC, respectively. For the 35 day data, ROC analysis revealed an AUC value of 0.770 (p = 0.05) versus 0.825 (p = 0.02) for PRM_ADC+_ and percent change in mean histogram ADC, respectively.

NAC is used as a standard treatment to improve surgical outcomes for patients with inoperable locally advanced breast cancer, and to improve breast conservation rates in patients with operable breast cancer. For such an approach to be optimally employed an accurate surrogate biomarker of therapeutic tumor response must be developed to identify patients unresponsive to NAC. DW-MR is one such imaging technique that has shown great promise at detecting tumor therapeutic response. A standard sequence on most MRI systems, DW-MR has found widespread use for diagnostic and prognostic application in the medical community [40,44,56,57]. The appeal of this technique is its high sensitivity to microenvironmental changes in living tissue that commonly occurs upon the onset or treatment of disease [58,59]. In addition, this MRI technique is inherently noninvasive, requiring no contrast administration. Preclinical work by our group and others as well as clinical studies have demonstrated the sensitivity of DW-MR for assessing early therapeutic response in breast cancer [26,27,35,36,60,61]. Recent advances in an analytical technique referred to as PRM has shown to improve the sensitivity of quantitative maps (i.e. ADC) at detecting even subtle therapy-induced changes within tumors [27,36–38,43,54,62–64]. In this study, ADC, the functional metric, was evaluated as a surrogate imaging biomarker using conventional summary statistical analysis and a voxel-based (PRM) approach for tumor response to NAC using subject data acquired from multiple clinical sites.

Through collaborative efforts of the Quantitative Imaging Network (http://imaging.cancer.gov/programsandresources/specializedinitiatives/qin) the reproducibility of DW-MR was evaluated by developing a thermal-controlled diffusion phantom as well as established clinical protocols for proper execution of the DW-MRI examination (ice water phantom and white papers) [52,57]. To date, therapeutic response using ADC measurements continues to be determined primarily using histogram derived whole-tumor summary statistics, where the mean of the ADC values are evaluated serially with disregard for any spatial dependence of the ADC measurements. In this study we observed that the mean ADC tumor values showed negligible variability between short interval serial examinations with |ΔADC|<0.1x10^-3^mm^2^/s. This is consistent with the deviation in ADC measurements observed from our repeatability analysis using our phantom where ADC values were typically between 1.05x10^-3^mm^2^/s and 1.1x10^-3^mm^2^/s. In contrast, ADC voxel values within the tumors showed substantially broad distributions, with standard deviations as large as 0.5x10^-3^mm^2^/s (Fig 2A and 4D). This is in contrast to our observations from the phantom study. ADC variability in the tumor tissue is attributed to the dependence of ADC voxel values to local microenvironments within the tumor (i.e. tumor cellularity, necrosis, edema and vasculature), which is unlike the homogeneous phantom that exhibits no spatial dependence in ADC. By propagating this error (i.e. standard deviation) we determined that the mean 95% CI of our serial ADC measurements (ΔADC) was approximately ±1x10^-3^mm^2^/s, with one subject generating a 95% CI as high as ±1.7x10^-3^mm^2^/s. It is this spatial variability, confounded with spatial variability in tumor response where local ADC simultaneously increases and decreases that attenuates the sensitivity of the whole-tumor statistical ADC measure. By way of spatial alignment of the serial ADC maps we are able to remove much of the variability in the data. Following this procedure, a step in our PRM method, we generated a mean 95% CI of ±0.45x10^-3^mm^2^/s (Fig 3C) which is significantly lower than the error observed in Fig 3B (p<0.0001 paired t-test).

We applied the PRM approach to serial ADC measurements from three separate prospective clinical trials to assess the sensitivity of PRM_ADC_ as a response metric to NAC in primary breast cancer patients. Our previous work and the work of others using various tumor types, both clinical and preclinical, have identified the relative volume of tumor that demonstrates a significant increase in ADC (PRM_ADC+_) as the most predictive of response [36–38,40,43,54]. Consistent with these findings we observed at the 35 day interval responders having significantly higher PRM_ADC+_ than non-responders (Fig 6B). Based on the literature where ADC values have been found to inversely correlate strongly with tumor cellularity, the results generated by our PRM method would in fact be in agreement with the “state” of the tumor. Recently, investigators have shown that voxel-based analysis of breast cancer DW-MRI along with dynamic contrast enhanced MR (DCE-MR) images can be used to provide spatial information related to response along with optimization of prognostic accuracy [65] furthering the concept that PRM improves diagnostic accuracy over whole-tumor histogram statistics [42].

There are some limitations in the current study that must be addressed. Test-retest data acquired from our subject population and the diffusion phantom were acquired using different MRI coils. Variations in coil designs may affect signal sensitivity and homogeneity, effecting noise levels in the ADC measurement. Also, if lesion location in the magnet bore relative to iso-center varied between the scans, then gradient nonlinearity may further increase variance. Nevertheless, this analysis illuminates the impact of tumor heterogeneity on quantitative ADC values as determined by DW-MRI. In addition, the repeatability analysis did not account for variations between centers and MRI platforms as all data was acquired at a single site on a single scanner. However, this analysis provides an indication of the robustness and variability of ADC measurements in this cohort of breast cancer subjects. Differences in study design, which include DW-MR sequence parameters examinations times, may have also resulted in an increase in the variability in the ADC measurements. Although pooling the data at the earlier time intervals was an option, due to differences in the study protocols we elected to treat the UM data independently from data acquired at Hull and NeoCOMICE. In contrast, both Hull and NeoCOMICE were pooled as the study designs were the same for these trials. Consequent to evaluating the data at their interval examination, patient numbers were relatively low. As such the predictive potential of the PRM method may be affected by type I error (false positive) and type II error (false negative). This is also confounded by differences in outcome measures between sites where UM used palpation, Hull and NeoCOMICE used RECIST 1.1. Nevertheless, the results presented in this study provide valuable information on the extent of variability in the ADC measurement and the ability of PRM to be used in multi-center imaging trials.

## Conclusion

Our findings support further development of ADC measurements with PRM analysis as a biomarker of early therapeutic response assessment of breast cancer patients undergoing NAC. The study also demonstrated the feasibility of performing a multi-site prospective analysis of DW-MRI as a predictive biomarker of NAC in breast cancer patients. In addition, we provide experimental results supporting the need for more sensitive analytical tools for evaluating ADC measurements such as PRM which also provides for spatial changes to be mapped versus histogram-based metrics. Future studies involving multi-center prospective clinical trials will require adequate quality assurance controls for uniformity in DW-MR sequences as well as consistent ADC measurements through the use of diffusion phantoms to qualify individual MR systems for conducting DW-MR scans. Moreover, multi-modal imaging metrics including hemodynamic information along with DW-MR metrics may add additional prognostic accuracy as was recently reported in a 28 patient NAC trial [34]. Overall, this study has provided approaches which can be implemented to ensure more unified and consistent data collection for improving cross comparison of DW-MR results between clinical sites. Overall, the results presented support the emerging role of DW-MR in the context of early treatment response assessment for breast cancer patients undergoing NAC but a larger multi-center prospective study is needed to confirm these findings.

## Supporting Information

S1 Table Summary of ADC and fDM imaging metrics for all subjects analyzed. (DOCX)
