# Supplementary material for: Multi-Site Clinical Evaluation of DW-MRI as a Treatment Response Metric for Breast Cancer Patients Undergoing Neoadjuvant Chemotherapy
Source: PLoS One. 2015 Mar 27;10(3):e0122151. doi: 10.1371/journal.pone.0122151 (PMC4376686; doi:10.1371/journal.pone.0122151)
Supplement: S1 Table — (DOCX) [file pone.0122151.s001.docx]

**S1 Table.** Summary of ADC and fDM imaging metrics for all subjects analyzed.
